# Microgravity-Induced Transcriptome Adaptation in Mouse Paraspinal *longissimus dorsi* Muscle Highlights Insulin Resistance-Linked Genes

**DOI:** 10.3389/fphys.2017.00279

**Published:** 2017-05-05

**Authors:** Guido Gambara, Michele Salanova, Stefano Ciciliot, Sandra Furlan, Martina Gutsmann, Gudrun Schiffl, Ute Ungethuem, Pompeo Volpe, Hanns-Christian Gunga, Dieter Blottner

**Affiliations:** ^1^Center of Space Medicine Berlin, Charité Universitätsmedizin BerlinBerlin, Germany; ^2^Institute of Anatomy, Charité Universitätsmedizin BerlinBerlin, Germany; ^3^Venetian Institute of Molecular Medicine, University of PadovaPadova, Italy; ^4^Department of Medicine, University of PadovaPadova, Italy; ^5^Institute of Neuroscience Consiglio Nazionale Delle RicerchePadova, Italy; ^6^Laboratory of Functional Genomics, Charité Universitätsmedizin BerlinBerlin, Germany; ^7^Dipartimento di Scienze Biomediche, University of PadovaPadova, Italy; ^8^Department for Physiology and Centre for Space Medicine, Charité Universitätsmedizin BerlinBerlin, Germany

**Keywords:** skeletal muscle, gene expression, microgravity, BION-M1, microarray, spaceflight, disuse, insulin resistance

## Abstract

Microgravity as well as chronic muscle disuse are two causes of low back pain originated at least in part from paraspinal muscle deconditioning. At present no study investigated the complexity of the molecular changes in human or mouse paraspinal muscles exposed to microgravity. The aim of this study was to evaluate *longissimus dorsi* adaptation to microgravity at both morphological and global gene expression level. C57BL/N6 male mice were flown aboard the BION-M1 biosatellite for 30 days (BF) or housed in a replicate flight habitat on ground (BG). Myofiber cross sectional area and myosin heavy chain subtype patterns were respectively not or slightly altered in *longissimus dorsi* of BF mice. Global gene expression analysis identified 89 transcripts differentially regulated in *longissimus dorsi* of BF vs. BG mice. Microgravity-induced gene expression changes of lipocalin 2 (Lcn2), sestrin 1(Sesn1), phosphatidylinositol 3-kinase, regulatory subunit polypeptide 1 (p85 alpha) (Pik3r1), v-maf musculoaponeurotic fibrosarcoma oncogene family protein B (Mafb), protein kinase C delta (Prkcd), Muscle Atrophy F-box (MAFbx/Atrogin-1/Fbxo32), and Muscle RING Finger 1 (MuRF-1) were further validated by real time qPCR analysis. In conclusion, our study highlighted the regulation of transcripts mainly linked to insulin sensitivity and metabolism in *longissimus dorsi* following 30 days of microgravity exposure. The apparent absence of robust signs of back muscle atrophy in space-flown mice, despite the overexpression of Atrogin-1 and MuRF-1, opens new questions on the possible role of microgravity-sensitive genes in the regulation of peripheral insulin resistance following unloading and its consequences on paraspinal skeletal muscle physiology.

## Introduction

Low back pain is a common concern for crew members in short or long term duration space flight. A complete retrospective study by Kerstman and co-workers showed that among 772 astronauts of the U.S space program, 382 were positive to low back pain (LBP), also called space adaptation back pain (SABP) (Kerstman et al., 2012). Back pain onset in the major part of the analyzed cases was reported during the first days of space flight (day 1–12; Wing et al., 1991). The pathophysiology of microgravity induced back pain has been previously investigated and it is likely to be discogenic and somatic. Microgravity abolishes the physiological loads of the spine with a consequent increase of body-length determined essentially by an intervertebral disk (IVD) swelling and an adaptation of the thoracic and lumbar spine curvature. Consequently the disk expansion can stimulate type IV mechanoreceptors contributing to lumbar back pain onset (Sayson and Hargens, 2008; Sayson et al., 2013).

Spinal stability in response to the gravity load is maintained through a stabilizing system assembled by a passive component (vertebrae, disks, and ligaments), an active component (muscle and tendons), and the nervous system. In this view, paraspinal muscles and tendons generate force required for the stability of the spine. The impairment of any component of this stabilizing system can induce spine instability, consequentially generating back pain (Panjabi, 1992a,b). The lack of gravity forces in the microgravity environment unavoidably impairs the muscle involved in posture and stability both at structural and functional level, potentially contributing directly, or indirectly to the onset of back pain. The intensity of SABP is usually mild or moderated, but it could also impact the activity of astronauts in space missions, increasing the risk of musculoskeletal injury, or other traumas (Scheuring et al., 2009). Moreover, it has also been shown that the risk of IVD herniation is increased in astronauts, particularly within the first year after landing, indicating the potentially risky effect of reloading after microgravity exposure and suggesting the application of specific behavioral/training protocols for re-adaptation to gravity in crew members in spaceflight (Johnston et al., 2010; Belavy et al., 2016). Therefore, further efforts are needed to better understand the effect of microgravity on spine and paraspinal muscles and to design effective new countermeasures able to prevent paraspinal myofascial and neuromuscular deconditioning.

Only few investigations were performed so far on the effect of microgravity on paraspinal skeletal muscles. LeBlanc et al. (1995, 2000) investigated the effect of spaceflight on different muscles of shuttle/Mir crew members, concluding that both short term (8 and 17-days) and medium term (16–28 weeks) flights significantly reduced the intrinsic back muscle volume. Recently, the functional cross sectional area of lumbar paraspinal muscle has been found significantly reduced in one astronaut following 6-months exposure to microgravity (Hides et al., 2016). Moreover, there is even less evidence about the effect of microgravity on the gene or protein expression in back muscle. For example, 14-days spaceflight reduced the number of type I muscle fibers, while the expression of 70 kDa heat shock protein, t complex polypeptide 1 and other mitochondrial proteins was upregulated in paraspinal rat muscle. On the other hand, myocyte-specific enhancing factor 2C and aldolase resulted downregulated following spaceflight (Yamakuchi et al., 2000). Recently, the E3-ligase MuRF-1 was found to be increased in *longissimus dorsi* of mice exposed to microgravity for 30 days (Mirzoev et al., 2014).

In 2013 male mice were flown onboard of the Russian BION-M1 biosatellite to evaluate the effect of 30 days microgravity exposure on different bio-parameters *in vivo* and *in vitro*. Ogneva I.V. and co-worker investigated changes in the cortical cytoskeleton structure in skeletal muscle of the BION-M1 flown mice, showing significant alteration in the content of alpha-actinin-1 and beta-actin respectively in *soleus* and *tibialis anterior* muscles (Ogneva et al., 2014). Among the BION-M1-based studies, it has been also observed a fiber type shift from slow to fast and a decrement of titin and nebulin proteins in *gastrocnemius* and *tibialis anterior* of flown mice (Ulanova et al., 2015).

The present study provides for the first time the global gene expression profile of *longissimus dorsi* in mice exposed to microgravity, which is central in further understanding of the structural, molecular and metabolic mechanism regulating vertebrate paraspinal muscle adaptation to spaceflight.

## Materials and methods

### Ethical approval

All procedures including animal were performed in agreement with the European Convention for the Protection of Vertebrate Animals used for Experimental and Other Scientific Purposes (Strasbourg, 18.03.1986). Institutional Animal Care and Use Committee (IACUC) of MSU Institute of Mitoengineering (Protocol 35, 1 November, 2012) and Biomedical Ethics Commission of Institute for Biomedical Problems (IBMP), Moscow (protocol 319, 4 April, 2013) approved the study protocol related to the BION-M1 mission.

### Animals

C57BL/N6 male mice (22–25 g) were obtained from the Animal Breeding Facility Branch of Shemyakin & Ovchinnikov Institute of Bioorganic Chemistry, Russia. Mice were transported to the animal facility of Moscow State University (Institute of Mitoengineering) for training and selection and 19–20 weeks old mice were used in all the experiments. In detail, mice were randomly divided in 3 groups: BION Flown (BF), mice to be flown aboard the BION-M1 biosatellite exposed for 30 days to microgravity, BION Ground (BG) mice housed for 30 days in the same biosatellite habitat on ground, and Flight Control (FC) mice housed in the animal facility during the duration of the spaceflight.

### Sample preparation and transportation

Mouse skeletal muscle tissue sampling of all the experimental groups (*longissimus dorsi* and tongue) and freezing was performed by our Russian partners on site (IMBP, Moscow, Russia, contract # Bion-M1/2013 between RF SRC-IMBP and the Charité Berlin, Germany). In particular post-flight tissue sampling was done within 12–14 h at the IMBP after landing in Kazakhstan. All frozen samples were delivered deeply frozen in dry ice and further processed in our laboratory.

### Immunohistochemistry and morphological analysis

Cryosections (8 μm thickness) of mouse *longissimus dorsi* (*n* = 2) were cut with a Leica cryostat (CM 1850, Leica Microsystems, Bensheim, Germany), mounted on charged slides, stored frozen at −80°C. Immunofluorescence with anti-MyHC isoform monoclonal antibodies were performed as previously described (Gambara et al., 2017): BA-D5 that recognizes type 1 MyHC isoform; SC-71 for type 2A MyHC isoform; BF-F3, for type 2B MyHC isoform (DSHB, Developmental Studies Hybridoma Bank, University of Iowa, Iowa City, IA). The sections were stained also using an anti-dystrophin antibody (Santa Cruz Biotechnology, Santa Cruz, CA) to visualize myofiber membranes. As secondary antibody, goat anti rabbit Alexa-635 and goat anti-mouse Alexa-555, goat anti-mouse Alexa-488 and goat anti-mouse Alexa 405-conjugated secondary antibody diluted to a final concentration of 1 μg/ml were used. For mouse-derived monoclonal primary and secondary antibodies, the MOM Ig blocking reagent (Vector Laboratories, Burlingame, CA, USA) was used to block mouse IgG background. Immunofluorescence were analyzed with an epifluorescence microscope (Axioplan; Zeiss, Oberkochen, Germany) equipped with a Cool Snap digital camera (Visitron Systems GmbH, Puchheim, Germany) and with 4-channel laser confocal microscope (TCS SP-8 with supersensitive HyD SP GaAsP detectors, Leica Microsystems, Germany). Digitized images were acquired with MetaVue software (Meta Series 7.5.6; system ID: 33693; Molecular Devices, Sunnyvale, CA, USA) and LAS X SP8 control software (Leica). Cross-sectional area (CSA) of the different myofiber types was semi-automatically measured by means of ImageJ 1.45 g (NIH, freeware imaging software).

### RNA extraction and sample target preparation

Total RNA was isolated from mouse *longissimus dorsi* (*n* = 5 for each group: BF, BG, and FC) and tongue (*n* = 3 for BF and BG; *n* = 2 for FC) muscles of each experimental group (BF, BG, and FC) by means of RNeasy micro Kit (Qiagen, Hilden, Germany). Frozen tissue samples were pulverized in liquid nitrogen and lysates were prepared in lysis buffer. Tissue lysate was centrifuged and the supernatant was used for RNA phenol/chloroform extraction. Single-step method of RNA isolation by acid guanidinium thiocyanate-phenol-chloroform extraction was performed. The aqueous layer was mixed with an equal volume of 70% ethanol and total RNA was extracted using RNeasy spin columns according to the manufacturer's protocol. RNA integrity was checked by 2100 Bioanalyzer (Agilent technologies, PA, USA). The amplification and labeling of the RNA samples were carried out according to the manufacturer's instructions (Affymetrix, Santa Clara, CA). Briefly, total RNA was quantified by and checked by analysis on a LabChip (BioAnalyzer, AGILENT Technologies, Santa Clara, CA). The GeneChip® 3′ IVT Express Protocol is based on the Eberwine or reverse transcription method (*in vitro* transcription, IVT). Starting from 100 nanogram total RNA, first strand DNA was synthesized, containing a T7 promotor sequence and then converted into a double-stranded DNA. The double strand DNA serves as template in the subsequent *in vitro* transcription (IVT) reaction. This amplification step generates Biotin labeled complementary RNA (cRNA). After cleanup the biotin-modified RNA was fragmented by alkaline treatment. Fifteen microgram of each cRNA sample was hybridized for 16 h at 45°C to an Affymetrix GeneChip Mouse 430A 2.0 Array. Arrays were washed and stained with streptavidin-phycoerythrin solutions using a fluidics station according to the protocols recommended by the manufacturer. Finally, probe arrays were scanned at 1.56-μm resolution using the Affymetrix GeneChip System confocal scanner 3000. Affymetrix Mouse Genome 430A 2.0 Array includes 22,600 probes sets to evaluate the expression level of more than 14,000 well-characterized mouse genes.

### Microarray data analysis and pathway analysis

Partek® Genomics Suite® 6.6 software was used for data analysis, applying Robust Multichip Average algorithm (RMA) for data normalization. To find differentially expressed genes, Analysis of Variance (ANOVA) was applied (*p* < 0.05). In the analysis of differentially regulated genes, a step up false discovery rate (FDR) of 5% was applied. Lists of differentially regulated genes included transcripts with fold changes major than 2 or minor than −2. Mouse Genome 430A 2.0 Array Probe Set Annotations was applied. Microarray data were uploaded in Gene Expression Omnibus (GEO) repository, accession number: GSE94381.

Gene ontology and pathway analysis were performed using the Functional Annotation Clustering module of DAVID v6.7 (The Database for Annotation, Visualization and Integrated Discovery). Gene enrichment were considered significant with an EASE score <0.05 (modified Fisher's exact test).

### Quantitative PCR validation

Quantitative PCR was performed by SYBR Green method from total RNA isolated from *longissimus dorsi* (*n* = 5) and tongue muscle (*n* = 3) of each experimental group as described above. Briefly, 400 ng of RNA were converted to cDNA by using random hexamers and SuperScript® VILO™ (Invitrogen) following the manufacturer's instructions. Specific primers for qPCR were already published (Sandona et al., 2012) or designed using Primer3 software (http://frodo.wi.mit.edu/, Whitehead Institute for Biomedical Research). Their thermodynamic specificity was determined using BLAST sequence alignment (NCBI) and vector NTI® software (Invitrogen). Oligonucleotide primers used are listed in Table S1. The reaction mix consists of 10 μl of 2x iQ SYBR Green Supermix (Bio-Rad), 0.3 pmol/μl primers, 8 ng of cDNA, and DNase/RNase-free water up to 20 μl. The PCR parameters were initial denaturation at 95°C for 30 s followed by 40 cycles of 10 s at 95°C and 30 s at the corresponding annealing temperature (55–59°C) for acquisition of fluorescence signal. A melting curve was generated by the iQ5 software (Biorad) following the end of the final cycle for each sample, by continuous monitoring the SYBR Green fluorescence throughout the temperature ramp from 65° to 99°C in 0.5 s increments. All samples were run in triplicate, in parallel for each individual muscle sample and simultaneously with RNA-negative controls. Cyclophilin A (*Ppia*), glyceraldehyde 3-phosphate dehydrogenase (*Gapdh*), pyruvate carboxylase (*Pcx*), and Beta-actin (*Actb*) were tested as candidate reference genes being the latter the most stable to normalize *Ct*-values by ΔCt method. Same data were obtained if *Ppia, Gapdh*, or *Pcx* were used as housekeeping controls (data not shown).

### Statistics

Data were analyzed by GraphPad software and expressed as means ± SE. Statistical differences between groups were determined by unpaired *t*-test. Differences were considered statistically significant at the *p* < 0.05 level of confidence.

## Results

### Morphological analysis showed moderate signs of muscle atrophy in *longissimus dorsi* of mice exposed to 30 days of microgravity

To assess whether microgravity exposure induced atrophy in *longissimus dorsi* of spaceflown mice (BF) compared to ground controls (BG and FC), haematoxylin-eosin (H.E.), and immunofluorescence analysis were performed. H.E. staining confirmed the absence of histopathological alterations, such as central nuclei, immune cell infiltration, or myofiber degeneration, in all experimental groups (BF, BG, and FC; data not shown).

**Figure 1:**
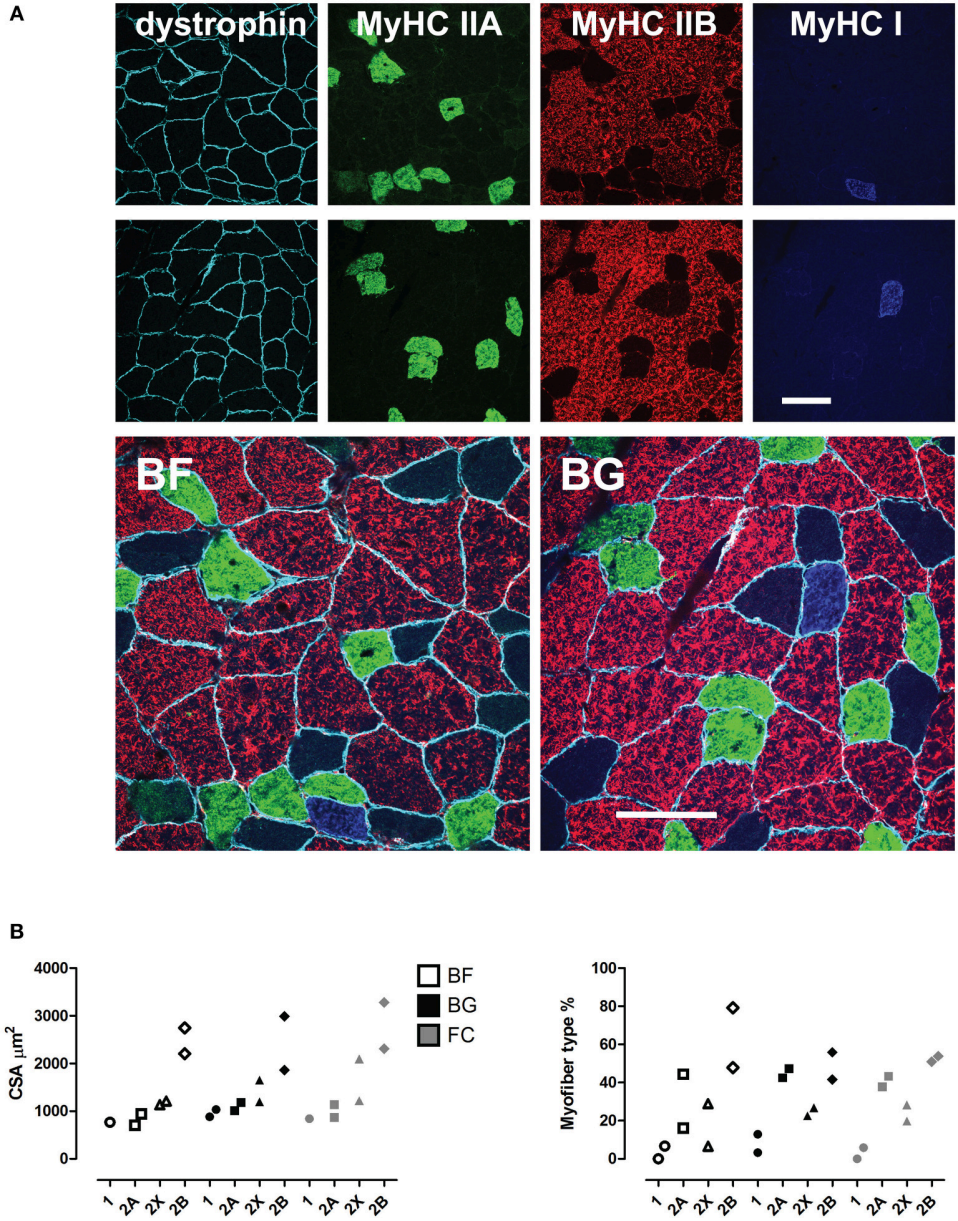
**Morphological analysis of mouse ***longissimus dorsi*** of space flown mice. (A)**, Immunohistochemical analysis (upper panel), and **(B)**, quantification of the myofiber cross sectional area (CSA, μm^2^) and type composition in *longissimus dorsi* back muscle of mice from the three experimental groups (BF, BG, and FC, each *n* = 2) shown as scatter plots (lower panel). Color-coded immuno-markers in **(A)**: dystrophin = turquoise; myosin heavy chain (MyHC) isoforms: IIA, green; IIB, red; IIX, negative; IA, blue. Scale bar: 100 μm.

To evaluate microgravity-induced myofiber cross sectional area (CSA) changes and phenotype shift (slow-to-fast) in skeletal muscle fibers, we performed multiple immunofluorescence staining using antibodies recognizing specific myosin heavy chain isoforms (MyHC I, 2A, 2B) and the sub sarcolemma marker dystrophin. As shown in Figure 1, no changes in CSA were observed in *longissimus dorsi* of spaceflown mice compared to ground controls. The comparison of the frequency distribution of CSAs in *longissimus dorsi* revealed no differences between the experimental groups (Figure S1A). Similar results were obtained calculating myofiber minimum Feret diameter (Figure S1B). In regard to myofiber type composition, we observed an increase in the percentage (Δ%) of fast 2B fibers in only one of the space-flown mice. Given the low number of animals analyzed (*n* = 2) for immunohistochemistry, these results suggest that 30 days of microgravity exposure may not be sufficient to induce robust CSA changes in mouse *longissimus dorsi*, though the onset of the phenotypic myofiber shift in the paraspinal muscle is already detectable.

### Microgravity-induced gene expression adaptation in *longissimus dorsi*

To analyse the adaptation to microgravity of the paraspinal muscle transcriptome, we performed an expression profile analysis of *longissimus dorsi* from space flown (BF) vs. ground control mice (BG: mice housed in the same biosatellite habitat on ground, and FC: mice housed in the animal facility). A total of 15 mouse *longissimus dorsi* (BF *n* = 5, BG *n* = 5, and FC *n* = 5) were analyzed. To identify genes differentially regulated in muscle of space flown mice, we compared the muscle transcriptome of BF vs. BG, FC vs. BG and BF vs. FC. The BF vs. BG and BF vs. FC comparisons (flown mice vs. ground controls) reflected the gene expression adaptation in skeletal muscle exposed to microgravity, while the comparison of the two ground controls (FC vs. BG) was needed to rule out possible gene expression changes originating from different housing conditions used in the present study protocol.

**Figure 2:**
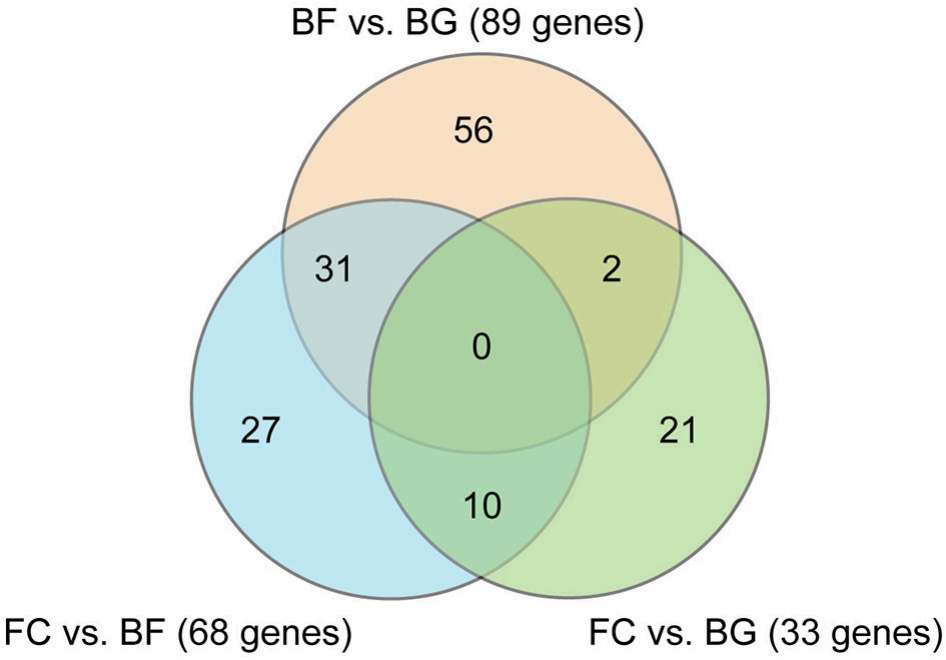
**Venn diagram showing the number of genes differentially regulated in ***longissimus dorsi*** comparing all the different experimental groups**. Venn diagram showing the number of genes differentially regulated comparing space flown (BF) with ground controls (BG and FC) in *longissimus dorsi* (each group *n* = 5). Comparisons between the three different experimental groups are shown (BF vs. BG, FC vs. BG, and BF vs. FC).

Affimetrix data were analyzed applying a False Discovery Rate (FDR) cut-off of 5% and a Fold Change (FC) cut-off < −2 and > 2. Venn diagram in Figure 2 shows the number of genes significantly differentially regulated in *longissimus dorsi* (BF vs. BG, FC vs. BG, and BF vs. FC). As expected, the higher number of differentially regulated genes were found comparing BF vs. BG (89 transcripts) and BF vs. FC (68 transcripts), indicating that microgravity exposure induced an adaptation of gene expression in *longissimus dorsi* of spaceflown mice (BF) compared to ground controls (BG and FC). On the other hand, the lower number of genes (33 genes) differentially regulated comparing the two ground controls (FC vs. BG), only 2 of which overlapping with transcripts found in BF vs. BG, indicated that the housing condition in the BION-M1 biosatellite on its own did not affect gene expression changes observed in muscle of flown mice compared with ground control (BG).

**Figure 3:**
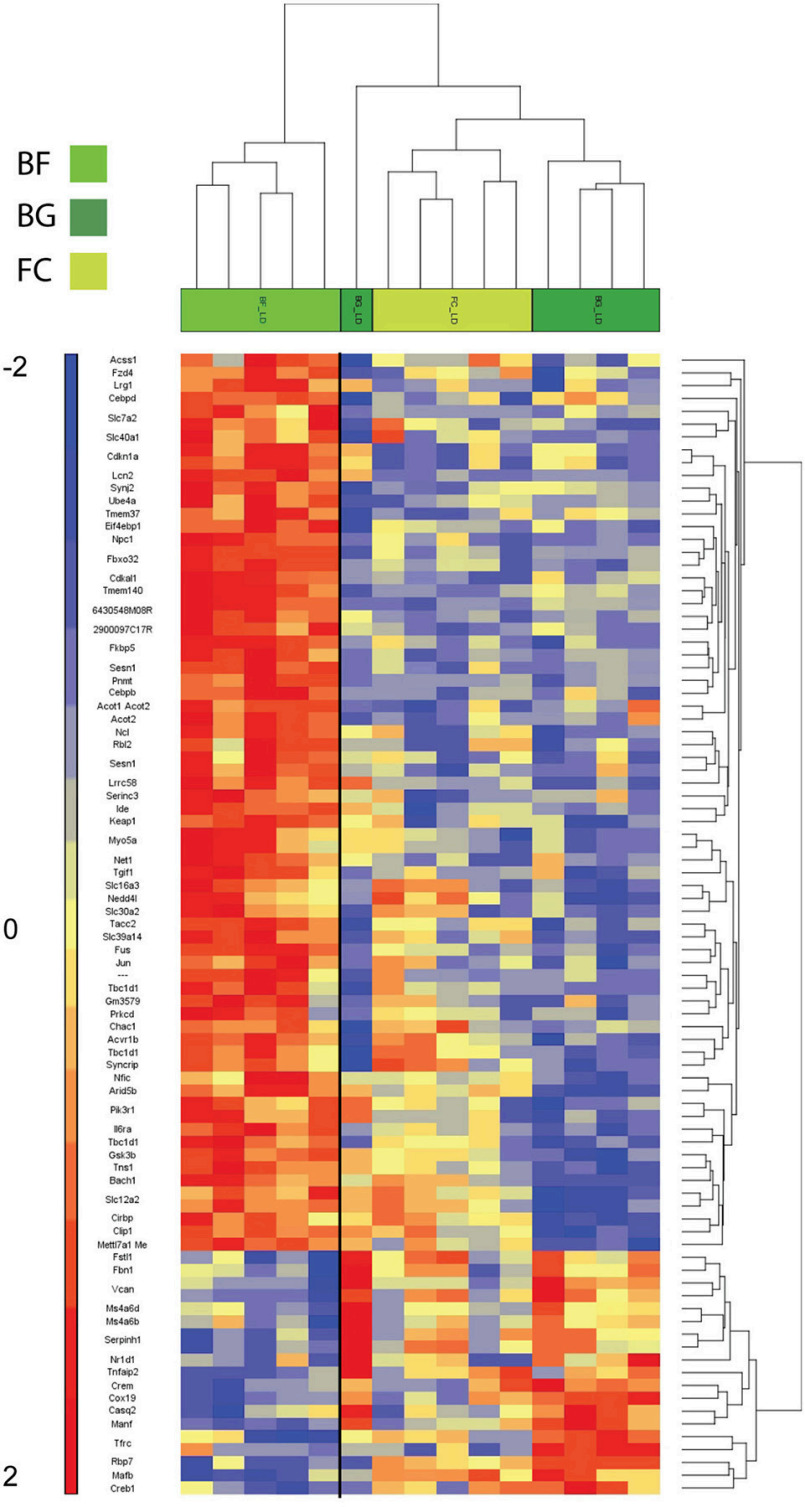
**Heat map showing genes differentially regulated in ***longissimus dorsi*** of BION-M1 space flown mice**. Two-way hierarchical clustering centered on BF vs. BG of mouse *longissimus dorsi* (each group *n* = 5). Differentially regulated genes meeting *FDR* < 0.05 and < −2 and > +2 fold change criteria are included in the heat map.

As shown in Figure 3, the two-way hierarchical clustering analysis centered on genes significantly differentially regulated in BF vs. BG revealed some similarity in the gene expression of the two ground controls (BG and FC) compared to *longissimus dorsi* muscles of microgravity exposed mice (BF) (Figure 3). Thus, BG and FC samples resulted in the same gene cluster arrangement.

**Figure 4:**
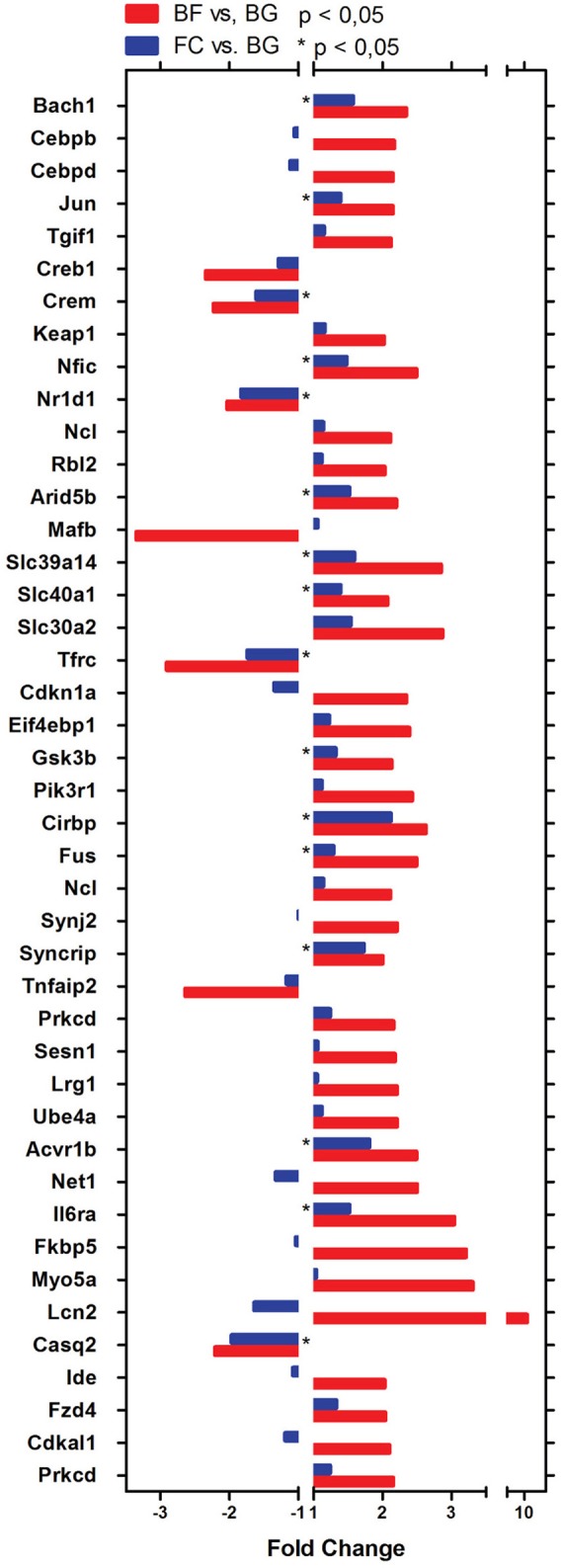
**Genes differentially regulated in ***longissimus dorsi*** of spaceflown mice identified by DAVID analysis**. Bar Chart representing genes linked to the main biological functions in BF vs. BG (red bars) and FC vs. BG (blue bars) comparison identified using DAVID analysis. All gene transcripts in BF vs. BG are significantly dysregulated (*p* < 0.05). For FC vs. BG transcripts significantly differentially regulated are indicated by ^*^p < 0.05.

One major aim of this study was to identify molecular players involved in *longissimus dorsi* adaptation process induced by exposure to microgravity environment. Thus, we focused the present analysis only on BF vs. BG comparison. Within the complete list of 89 genes differentially regulated in BF vs. BG (Table 1), the expression of 19 genes was downregulated, while 70 genes were upregulated. Next we performed gene ontology (GO) analysis to identify genes specifically linked to biological functions or molecular pathways. Database for Annotation, Visualization and Integrated Discovery (DAVID) analysis identified genes linked to four main functional clusters (Table 2): transcription regulation, transporter activity, ErbB signaling pathway, RNA recognition motif and other genes of interest (classified as other/unknown in Table 2). All the genes included in these functional clustering were significantly and differentially regulated in BF vs. BG, while only few of such genes were differentially regulated significantly compared to the two ground controls (BG vs. FC) or with a fold change < −2 or > 2, thus confirming that the observed changes in gene expression were specifically induced by microgravity exposure (Figure 4).

**Table 1:** *****Longissimus dorsi*** transcriptome adaptation to microgravity exposure**.

			**BF vs. BG**		**FC vs. BG**		**BF vs. FC**	
**Probeset ID**	**Entrez gene**	**Gene symbol**	***p*-value**	**Fold-change**	***p*-value**	**Fold-change**	***p*-value**	**Fold-change**
1451716_at	16658	Mafb	3.70E-05	−3.35	0.80239	1.06	2.22E-05	−3.54
1452661_at	22042	Tfrc	0.000274	−2.91	0.030046	−1.74	0.042065	−1.67
1419599_s_at	68774	Ms4a6d	0.002668	−2.67	0.011754	−2.20	0.498784	−1.21
1438855_x_at	21928	Tnfaip2	0.001161	−2.64	0.545691	−1.17	0.004421	−2.26
1427256_at	13003	Vcan	2.86E-05	−2.49	0.019029	−1.52	0.00704	−1.64
1460208_at	14118	Fbn1	0.004033	−2.36	0.070378	−1.65	0.182047	−1.43
1421583_at	12912	Creb1	0.004128	−2.34	0.329644	−1.29	0.033861	−1.81
1421694_a_at	13003	Vcan	1.60E-05	−2.30	0.000262	−1.90	0.192589	−1.21
1428112_at	74840	Manf	4.68E-06	−2.24	0.00036	−1.72	0.047102	−1.30
1449037_at	12916	Crem	0.000264	−2.23	0.014286	−1.61	0.081056	−1.38
1422529_s_at	12373	Casq2	0.000149	−2.21	0.000655	−1.97	0.498468	−1.12
1418826_at	69774	Ms4a6b	0.003911	−2.18	0.022244	−1.80	0.422954	−1.21
1449461_at	63954	Rbp7	0.004209	−2.18	0.402653	−1.22	0.025728	−1.78
1450843_a_at	12406	Serpinh1	0.000299	−2.15	0.065647	−1.40	0.020373	−1.54
1448259_at	14314	Fstl1	0.001288	−2.14	0.322366	−1.22	0.011567	−1.75
1422967_a_at	22042	Tfrc	0.00107	−2.12	0.00023	−2.43	0.484369	1.15
1456733_x_at	12406	Serpinh1	0.001236	−2.03	0.127213	−1.34	0.036967	−1.51
1426464_at	217166	Nr1d1	0.003783	−2.03	0.010649	−1.83	0.63518	−1.11
1434923_at	68033	Cox19	5.50E-07	−2.01	0.000198	−1.53	0.007355	−1.31
1422768_at	56403	Syncrip	1.62E-05	2.00	0.000225	1.73	0.217858	1.16
1423269_a_at	83814	Nedd4l	0.000355	2.01	0.005813	1.64	0.213553	1.22
1450747_at	50868	Keap1	2.91E-05	2.02	0.255532	1.16	0.000338	1.74
1418146_a_at	19651	Rbl2	0.000403	2.03	0.480517	1.12	0.001918	1.80
1434150_a_at	70152 /// 393082	Mettl7a1 Mettl7a2	1.40E-05	2.03	0.022879	1.34	0.00268	1.51
1453988_a_at	15925	Ide	0.000412	2.03	0.638871	−1.08	0.000149	2.20
1419301_at	14366	Fzd4	0.000254	2.04	0.080389	1.33	0.013902	1.53
1432646_a_at	347740	2900097C17Rik	1.08E-06	2.05	0.342346	−1.10	2.01E-07	2.26
1417061_at	53945	Slc40a1	5.04E-05	2.07	0.025295	1.39	0.009503	1.48
1423953_at	68916	Cdkal1	1.93E-06	2.10	0.117263	−1.19	1.13E-07	2.49
1415773_at	17975	Ncl	2.48E-05	2.11	0.32501	1.14	0.000201	1.85
1449405_at	21961	Tns1	3.17E-06	2.11	0.020294	1.32	0.000568	1.59
1449005_at	80879	Slc16a3	0.001438	2.11	0.054044	1.50	0.102055	1.40
1431213_a_at	100041932	Gm3579	0.001169	2.12	0.408605	1.18	0.007302	1.80
1422286_a_at	21815	Tgif1	2.34E-05	2.12	0.309256	1.15	0.000204	1.85
1451020_at	56637	Gsk3b	8.27E-06	2.13	0.036157	1.32	0.000913	1.62
1417611_at	170706	Tmem37	0.000781	2.13	0.654402	−1.09	0.000293	2.32
1422997_s_at	26897 /// 171210	Acot1 /// Acot2	0.000628	2.14	0.108529	−1.36	1.84E-05	2.92
1417409_at	16476	Jun	5.39E-05	2.15	0.035148	1.39	0.007188	1.55
1423233_at	12609	Cebpd	0.002984	2.15	0.601471	−1.12	0.00094	2.41
1422847_a_at	18753	Prkcd	0.000377	2.16	0.233831	1.24	0.005448	1.74
1431320_a_at	17918	Myo5a	0.00029	2.17	0.792485	1.05	0.000514	2.07
1427844_a_at	12608	Cebpb	6.68E-06	2.17	0.640688	−1.06	2.73E-06	2.30
1433711_s_at	140742	Sesn1	2.03E-07	2.18	0.564539	1.06	5.49E-07	2.06
1425745_a_at	57752	Tacc2	4.48E-07	2.18	0.003064	1.41	0.000366	1.55
1456080_a_at	26943	Serinc3	0.001568	2.18	0.242556	−1.29	0.000117	2.80
1452082_at	234797	6430548M08Rik	3.14E-08	2.18	0.046032	−1.19	1.52E-09	2.60
1416617_at	68738	Acss1	0.000348	2.19	0.021219	1.56	0.072344	1.40
1454699_at	140742	Sesn1	6.86E-05	2.19	0.577294	−1.09	2.16E-05	2.39
1420973_at	71371	Arid5b	1.13E-05	2.20	0.004402	1.52	0.011325	1.44
1431697_at	20975	Synj2	1.26E-05	2.21	0.989386	−1.00	1.23E-05	2.21
1417290_at	76905	Lrg1	0.000307	2.21	0.799724	1.05	0.000533	2.11
1425384_a_at	140630	Ube4a	1.40E-06	2.21	0.316461	1.12	9.68E-06	1.98
1427131_s_at	320184	Lrrc58	4.95E-05	2.22	0.559443	1.09	0.000171	2.03
1419448_at	57915	Tbc1d1	7.25E-07	2.23	0.004296	1.41	0.000474	1.57
1438931_s_at	140742	Sesn1	0.000307	2.24	0.501495	1.13	0.001353	1.98
1455892_x_at			1.94E-05	2.29	0.284654	1.17	0.000188	1.96
1425514_at	18708	Pik3r1	0.001526	2.30	0.342072	1.24	0.012489	1.85
1426446_at	234797	6430548M08Rik	3.59E-06	2.32	0.302904	−1.14	5.30E-07	2.65
1424638_at	12575	Cdkn1a	0.000231	2.34	0.118787	−1.35	8.16E-06	3.16
1449311_at	12013	Bach1	6.06E-06	2.34	0.003171	1.57	0.007878	1.49
1423086_at	18145	Npc1	4.63E-08	2.38	0.225594	1.12	3.56E-07	2.11
1417563_at	13685	Eif4ebp1	4.14E-07	2.39	0.071658	1.23	1.42E-05	1.93
1419447_s_at	57915	Tbc1d1	1.51E-05	2.41	0.000777	1.82	0.075419	1.32
1425515_at	18708	Pik3r1	0.000605	2.43	0.605949	1.12	0.00189	2.17
1421679_a_at	12575	Cdkn1a	0.00021	2.44	0.104343	−1.39	6.51E-06	3.38
1450703_at	11988	Slc7a2	2.10E-05	2.48	0.818778	1.04	3.36E-05	2.39
1417623_at	20496	Slc12a2	0.000126	2.49	0.007085	1.76	0.078497	1.41
1433725_at	11479	Acvr1b	6.23E-06	2.49	0.000635	1.81	0.037663	1.38
1422565_s_at	18029	Nfic	8.26E-05	2.49	0.040319	1.48	0.009631	1.68
1451285_at	233908	Fus	1.61E-07	2.49	0.029668	1.29	1.14E-05	1.93
1421321_a_at	56349	Net1	0.000152	2.50	0.153148	−1.33	7.40E-06	3.31
1448780_at	20496	Slc12a2	8.91E-05	2.51	0.00241	1.90	0.142167	1.32
1416332_at	12696	Cirbp	4.12E-05	2.63	0.000551	2.12	0.234157	1.24
1422648_at	11988	Slc7a2	5.22E-05	2.66	0.049873	1.47	0.004744	1.81
1451382_at	69065	Chac1	0.000401	2.66	0.009432	1.92	0.16174	1.39
1419446_at	57915	Tbc1d1	5.79E-06	2.72	0.028562	1.45	0.000788	1.88
1416125_at	14229	Fkbp5	4.92E-07	2.81	0.158763	1.21	7.24E-06	2.31
1427035_at	213053	Slc39a14	3.52E-07	2.85	0.002438	1.59	0.000339	1.79
1427339_at	230810	Slc30a2	0.000124	2.87	0.060195	1.54	0.009373	1.87
1424354_at	68487	Tmem140	6.50E-07	2.94	0.549542	−1.09	2.29E-07	3.20
1422996_at	171210	Acot2	2.27E-05	3.01	0.521089	−1.13	6.29E-06	3.41
1452416_at	16194	Il6ra	1.63E-07	3.04	0.005239	1.52	6.10E-05	2.00
1425060_s_at	56430	Clip1	3.43E-05	3.20	0.005692	1.94	0.028004	1.65
1448231_at	14229	Fkbp5	1.03E-06	3.21	0.806432	−1.04	6.61E-07	3.33
1419754_at	17918	Myo5a	0.000219	3.31	0.888517	1.04	0.000296	3.19
1448747_at	67731	Fbxo32	3.64E-09	4.50	0.121024	1.25	3.85E-08	3.60
1450606_at	18948	Pnmt	6.42E-08	4.56	0.61962	1.09	1.44E-07	4.19
1417522_at	67731	Fbxo32	1.23E-07	5.14	0.525366	1.13	3.60E-07	4.55
1427747_a_at	16819	Lcn2	1.02E-07	11.06	0.089068	−1.64	6.76E-09	18.13

*Complete list of genes found differentially regulated in longissimus dorsi of BF vs. BG mice, meeting FDR < 0.05 and fold change < −2 and > +2 criteria*.

**Table 2:** **Genes differentially regulated in ***longissimus dorsi*** of spaceflown mice linked to main functional clusters identified by DAVID**.

			**LD**					
			**BF vs. BG**		**FC vs. BG**		**BF vs. FC**	
	**Entrez gene**	**Gene symbol**	***p*-value**	**FC**	***p*-value**	**FC**	***p*-value**	**FC**
Transcription regulation	12013	Bach1	6.06E-06	2.34	0.003171	1.57	0.007878	1.49
	12608	Cebpb	6.68E-06	2.17	0.640688	−1.06	2.73E-06	2.30
	12609	Cebpd	0.00298405	2.15	0.601471	−1.12	0.00094	2.41
	16476	Jun	5.39E-05	2.15	0.035148	1.39	0.007188	1.55
	21815	Tgif1	2.34E-05	2.12	0.309256	1.15	0.000204	1.85
	12912	Creb1	0.00412805	−2.34	0.329644	−1.29	0.033861	−1.81
	12916	Crem	0.00026409	−2.23	0.014286	−1.61	0.081056	−1.38
	50868	Keap1	2.91E-05	2.02	0.255532	1.16	0.000338	1.74
	18029	Nfic	8.26E-05	2.49	0.040319	1.48	0.009631	1.68
	217166	Nr1d1	0.00378344	−2.03	0.010649	−1.83	0.63518	−1.11
	17975	Ncl	2.48E-05	2.11	0.32501	1.14	0.000201	1.85
	19651	Rbl2	0.00040274	2.03	0.480517	1.12	0.001918	1.80
	71371	Arid5b	1.13E-05	2.20	0.004402	1.52	0.011325	1.44
	16658	Mafb	3.70E-05	−3.35	0.80239	1.06	2.22E-05	−3.54
Transporter activity	213053	Slc39a14	3.52E-07	2.85	0.002438	1.59	0.000339	1.79
	53945	Slc40a1	5.04E-05	2.07	0.025295	1.39	0.009503	1.48
	230810	Slc30a2	0.00012372	2.87	0.060195	1.54	0.009373	1.87
	22042	Tfrc	0.00027448	−2.91	0.030046	−1.74	0.042065	−1.67
ErbB signaling pathway	16476	Jun	5.39E-05	2.15	0.035148	1.39	0.007188	1.55
	12575	Cdkn1a	0.00023117	2.34	0.118787	−1.35	8.16E-06	3.16
	13685	Eif4ebp1	4.14E-07	2.39	0.071658	1.23	1.42E-05	1.93
	56637	Gsk3b	8.27E-06	2.13	0.036157	1.32	0.000913	1.62
	18708	Pik3r1	0.000605	2.43	0.605949	1.12	0.00189	2.17
RRM (RNA recognition motif)	12696	Cirbp	4.12E-05	2.63	0.000551	2.12	0.234157	1.24
	233908	Fus	1.61E-07	2.49	0.029668	1.29	1.14E-05	1.93
	17975	Ncl	2.48E-05	2.11	0.32501	1.14	0.000201	1.85
	20975	Synj2	1.26E-05	2.21	0.989386	−1.00	1.23E-05	2.21
	56403	Syncrip	1.62E-05	2.00	0.000225	1.73	0.217858	1.16
Other/unknown	21928	Tnfaip2	0.00116133	−2.64	0.545691	−1.17	0.004421	−2.26
	18753	Prkcd	0.00037714	2.16	0.233831	1.24	0.005448	1.74
	140742	Sesn1	2.03E-07	2.18	0.564539	1.06	5.49E-07	2.06
	76905	Lrg1	0.00030677	2.21	0.799724	1.05	0.000533	2.11
	140630	Ube4a	1.40E-06	2.21	0.316461	1.12	9.68E-06	1.98
	11479	Acvr1b	6.23E-06	2.49	0.000635	1.81	0.037663	1.38
	56349	Net1	0.00015206	2.50	0.153148	−1.33	7.40E-06	3.31
	16194	Il6ra	1.63E-07	3.04	0.005239	1.52	6.10E-05	2.00
	14229	Fkbp5	1.03E-06	3.21	0.806432	−1.04	6.61E-07	3.33
	17918	Myo5a	0.00021875	3.31	0.888517	1.04	0.000296	3.19
	16819	Lcn2	1.02E-07	11.06	0.089068	−1.64	6.76E-09	18.13
	12373	Casq2	0.00014932	−2.21	0.000655	−1.97	0.498468	−1.12
	15925	Ide	0.00041213	2.03	0.638871	−1.08	0.000149	2.20
	14366	Fzd4	0.00025444	2.04	0.080389	1.33	0.013902	1.53
	68916	Cdkal1	1.93E-06	2.10	0.117263	−1.19	1.13E-07	2.49
	18753	Prkcd	0.00037714	2.156	0.233831	1.24	0.005448	1.74

*List of genes linked to five different gene clusters (transcriptional regulation, transported regulation, ErbB signaling pathway, RNA recognition motif, and other/unknown) differentially regulated in longissimus dorsi of BF vs. BG mice. Gene transcripts meeting FDR < 0.05 and fold change < −2 and > +2 criteria are included*.

### Real time PCR validation of microgravity-induced gene expression changes in *longissimus dorsi*

**Figure 5:**
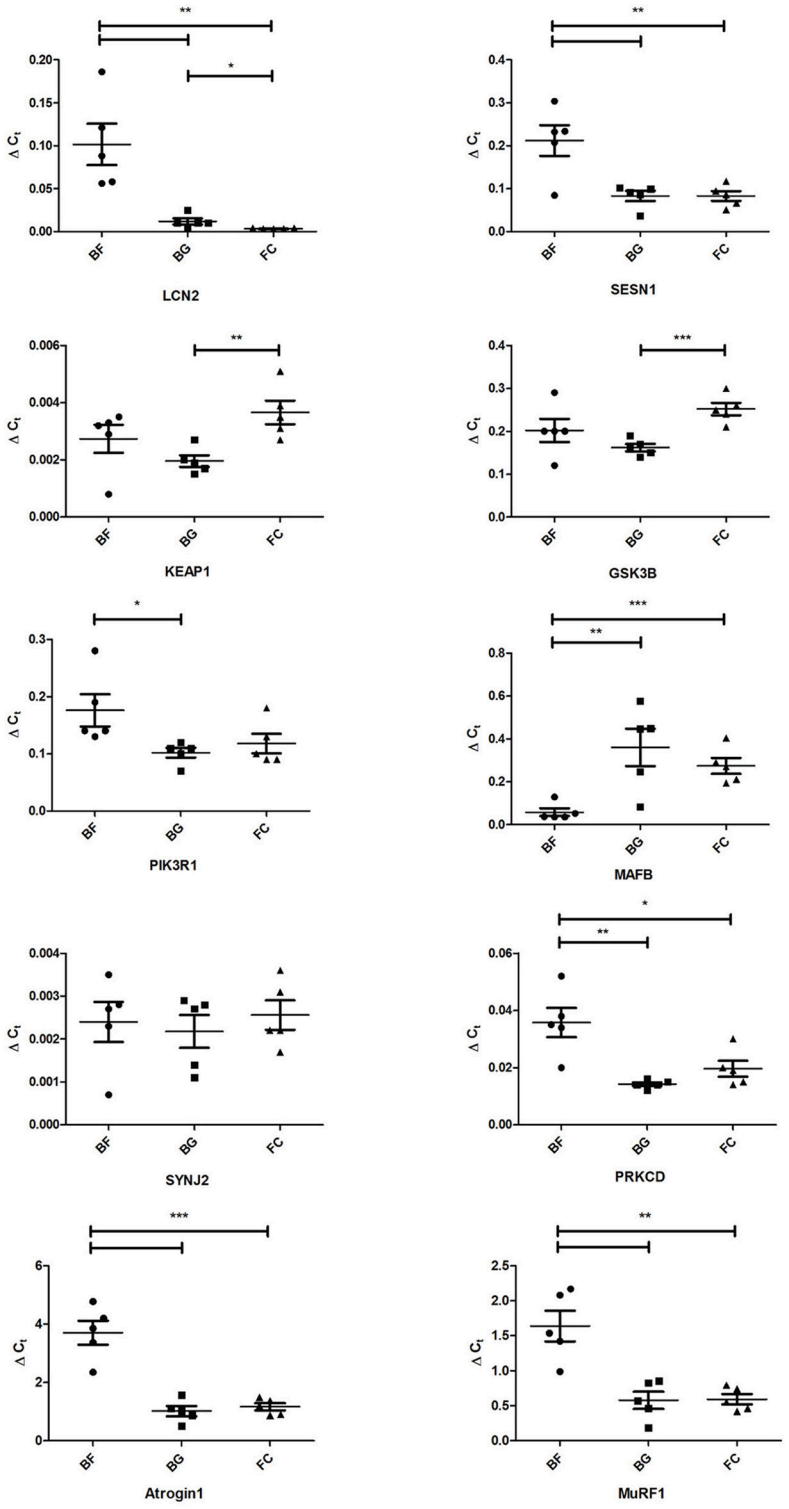
**Real time qPCR analysis of selected genes differentially regulated in ***longissimus dorsi*** of space-flown mice compared to ground controls**. Expression levels of lipocalin 2 (Lcn2), sestrin 1(Sesn1), kelch-like ECH-associated protein 1(Keap1), glycogen synthase kinase 3 beta (Gsk3b), phosphatidylinositol 3-kinase, regulatory subunit polypeptide 1 (p85 alpha) (Pik3r1), v-maf musculoaponeurotic fibrosarcoma oncogene family protein B (Mafb), synaptojanin 2 (Synj2), protein kinase C delta (Prkcd), Muscle Atrophy F-box (Atrogin-1), and Muscle RING Finger 1 (MuRF-1) were evaluated by real-time quantitative PCR in *longissimus dorsi* of space-flown mice (BF, *n* = 5) and ground controls (BG and FC, each *n* = 5). Graph shows ΔC_t_ ± SEM; ^***^p < 0.0009, ^**^p < 0.0095, and ^*^p < 0.05.

Quantitative real time PCR (qPCR) was performed to validate gene expression changes observed in *longissimus dorsi* following 30 days spaceflight (*n* = 5 for each group: BF, BG, and FC). The choice of genes to be validated was essentially centered on their *p*-values, fold changes (> 2.0 or < −2.0), and the potential association with skeletal muscle pathophysiology. Due to the low amount of tissue used for RNA extraction, we evaluated only the expression of 10 genes by qPCR in flown mice (BF) compared to ground controls (BG and FC): lipocalin 2 (Lcn2), sestrin 1(Sesn1), kelch-like ECH-associated protein 1(Keap1), glycogen synthase kinase 3 beta (Gsk3b), phosphatidylinositol 3-kinase, regulatory subunit polypeptide 1 (p85 alpha) (Pik3r1), v-maf musculoaponeurotic fibrosarcoma oncogene family protein B (Mafb), synaptojanin 2 (Synj2), protein kinase C delta (Prkcd), Muscle Atrophy F-box (MAFbx/Atrogin-1/Fbxo32), and Muscle RING Finger 1 (MuRF-1). Table S1 includes the sequence of the primer used in qPCR analysis. Four housekeeping genes were selected as reference to calculate the delta Ct of the selected genes: *Actb*, beta actin; *Ppia*, cyclophilin A; *Gapdh*, glyceraldehyde-3-phosphate dehydrogenase and *Pcx*, pyruvate carboxylase. PCR data normalized using Actb confirmed that Lcn2, Sesn1, Pik3r1, Mafb, Prkcd, Atrogin-1, and MuRF-1 were differentially regulated significantly in *longissimus dorsi* of BF compared to BG mice (Lcn2 *p* = 0.0062, Sesn1 *p* = 0.0090, Pik3r1 *p* = 0.036, Mafb *p* = 0.0094, Prkcd *p* = 0.0030, Atrogin-1 *p* = 0.0003, and MuRF-1 *p* = 0.0029), confirming the reliability of the Affymetrix data analysis (Figure 5). Identical results were obtained using Gapdh, Ppia, and Pcx as housekeeping genes (Data not shown).

### Tongue muscle as in-flight negative control of microgravity transcriptome adaptation

**Figure 6:**
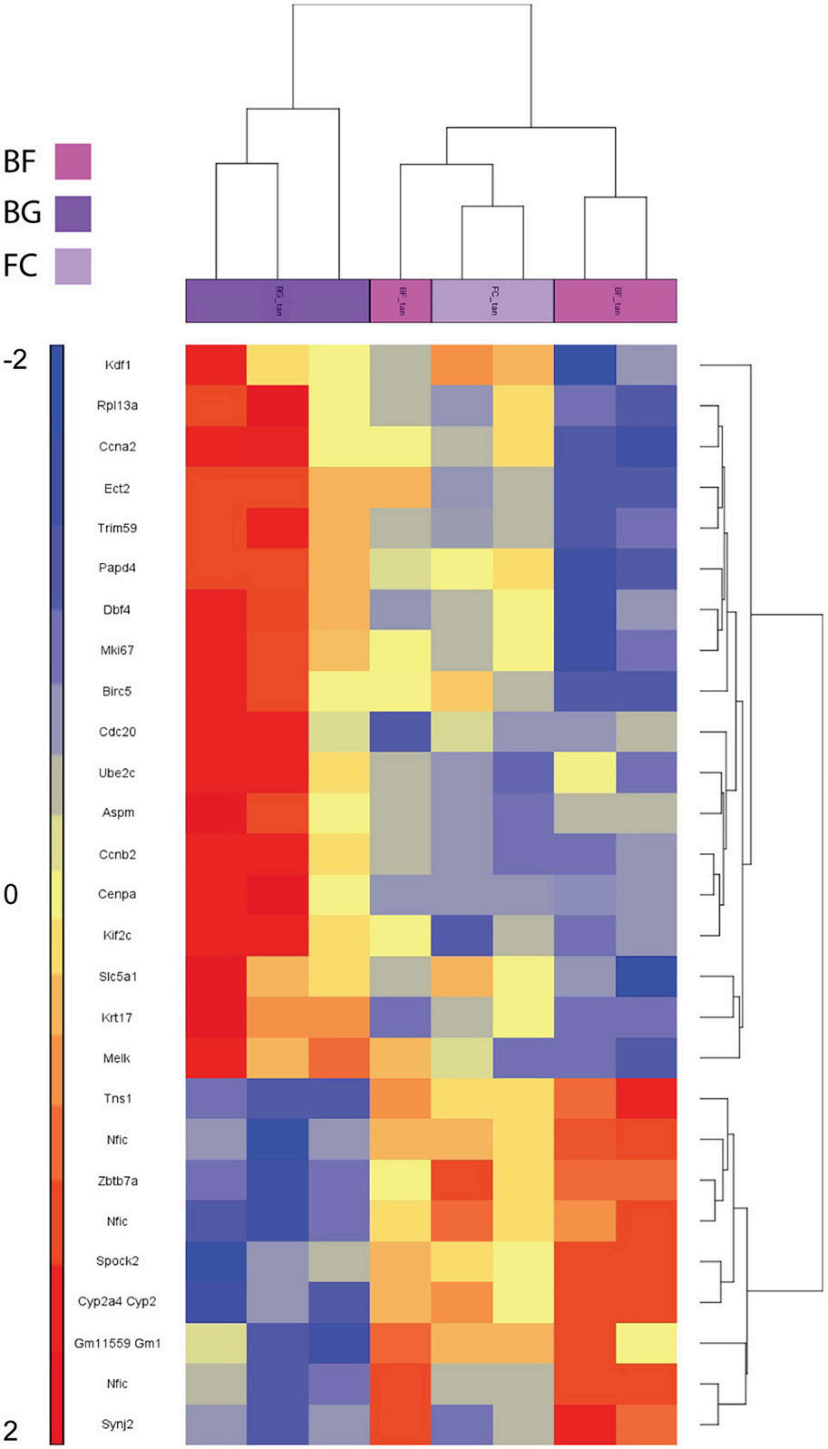
**Heat map visualizing genes differentially regulated in the tongue of BION-M1 space flown mice**. Two way hierarchical clustering centered on BF vs. BG of mouse tongue muscles (*n* = 3 for BF and BG; *n* = 2 for FC). Differentially regulated genes meeting *FDR* < 0.05 and fold change < −2 and > +2 criteria are included in the heat map.

To investigate whether microgravity exposure affected gene expression also in muscles that are constantly “activity loaded” also in spaceflight, we analyzed the transcriptome of mouse tongue from spaceflown mice (BF) compared to ground control (BG). Affymetrix analysis of BF vs. BG (applying a *FDR* <5% and fold change < −2 and > 2 cut-off) showed that only 27 genes were differentially regulated in the tongue muscle of space-flown mice. Table S2 shows the complete list of differentially regulated genes in tongue. The two way hierarchical clustering analysis centered on genes differentially regulated in BF vs. BG showed similarity in the gene expression of the BF and FC groups (Figure 6), suggesting that in this particular non-appendicular visceral skeletal muscle gene expression regulation has a relatively low sensitivity to microgravity exposure compared to appendicular or somatic skeletal muscle in murine vertebrates.

## Discussion

Our study investigated for the first time paraspinal muscle adaptation in 30 days space-flown mice both at morphological and gene expression level. At present, only few studies analyzed the effect of microgravity exposure on paraspinal skeletal muscle, and to our knowledge no study actually quantified the changes in either myofiber cross sectional area or fiber type composition neither in spaceflight crew member or space-flown rodent paraspinal back muscles. So far human studies on lumbar back muscle are mostly based on clinical imaging (magnetic resonance imaging) and physiological measurements (e.g., strength test). On the other hand, the effects on human paraspinal muscle deconditioned by the spaceflight analog bed rest have been already investigated to test efficacy of countermeasures able to prevent or reduce the impact of extended disuse or unloading of the spine. In a 60-days 6° head-down tilt (HDT) bed rest study, resistive exercise with or without superimposed whole body vibration reduced the whole muscle cross sectional area (CSA) loss observed in different paraspinal functional muscle groups such as lumbar *multifidus* (medial group), lumbar *erector spinae* (lateral group) and the anterior and medial hip group with the *psoas* (Belavy et al., 2010). Moreover, it has been recently reported that lower body negative pressure (LBNP) treadmill and flywheel significantly reduced total lumbar paraspinal muscle loss (76% represented by erector spinae) induced by 60 days of 6° HDT bed rest in women (Holt et al., 2016). Unfortunately, paraspinal back muscle biopsies, which would allow a more accurate view on disuse-induced structural and molecular tissue changes, may not be feasible from bed rest participants or even crew in Space because of two major constraints: firstly, the paraspinal back muscle shows some anatomical and functional complexity, for example subdivision into several muscular subgroups (e.g., medial deeper and lateral superficial columns), and secondly, there are obvious ethical constraints and risks of invasive biopsy sampling procedure on the back muscles of the participant's trunk staying supine for longer periods in bed rest.

However, the results reported in this study are based on the quantification of myofibers CSA and type composition of *longissimus dorsi* tissue samples from mice exposed to microgravity for as long as 30 days, showing that spaceflight only moderately affected the myofiber CSA and only slightly increased the amount of type 2B myofibers in *longissimus dorsi* of space-flown mice compared to ground controls. These results suggest that, despite elevated levels of Atrogin-1 and MuRF-1 found in muscle of space-flown mice, robust morphological changes distinctive of paraspinal muscle deconditioning are likely just to be set on in mice exposed for 30 days to microgravity. Our present data on mice partially disagree with those of Yamakuchi and co-workers, who described a “qualitative” decrease in the number and volume of type 1 MyHC positive myofibers in paraspinal rat muscle exposed to microgravity for 14 days (Yamakuchi et al., 2000). On the other hand, we expected to find some lower effects of microgravity adaptation in mouse *longissimus dorsi* compared to the robust changes previously observed in crewmembers after spaceflight or long term bed rest participants, considering the obvious differences in paraspinal muscle loading between bipedal humans and four-legged vertebrates on ground, and in particular during body movement adaptations of both species observed in the microgravity environment. Moreover, another possible source of discrepancy between our results and human studies might originate by the anatomical complexity of back muscles and the type of analysis performed. The major part of the human studies analyzing paraspinal muscle deconditioning focuses on imaging analysis (MRI) of functional groups of muscle in the lumbar region, such as the *erector spinae* (including components of different muscles such as *multifidus, longissimus dorsi, iliocostalis*, and *spinalis dorsi* (Belavy et al., 2010; Holt et al., 2016).

Though, the apparent lack of an atrophic structural phenotype in mouse *longissimus dorsi* (i.e., almost constant myofiber CSA with little slow-to-fast myofiber shift) was also confirmed by the global gene expression and gene ontology (GO) analysis, by which no genes linked to the sarcomere structure were found differentially regulated in space-flown mice. Surprisingly, transcripts coding for two ubiquitin ligases, Atrogin-1 and MuRF-1, involved in skeletal muscle atrophy (Bodine et al., 2001) were upregulated in space-flown mice compared with ground controls. Since Atrogin-1 and MuRF-1 expression levels have never been previously investigated in *longissimus dorsi* following unloading, different mechanisms could explain the overexpression of these two ubiquitin ligases without robust signs of muscle atrophy. For example, it is known that in rodent skeletal muscles the over-expression of transcripts coding for Atrogin-1 and MuRF-1 occurs relatively early following unloading and decreases back to baseline level within 15 days (Bodine et al., 2001; Bodine and Baehr, 2014). Thus, these data suggest that the expression of these two genes, usually considered an early event in the onset of the atrophic phenotype, is instead a relatively late event (30 days) in *longissimus dorsi* of space flown mice. Another possible explanation could involve a muscle specific mechanism regulating the translation of both transcripts. For example, it has been previously shown that miR-23 a suppresses the translation of both MAFbx/atrogin-1 and MuRF-1 in a 3_-UTR-dependent manner (Wada et al., 2011). Unfortunately, in the absence of time course experiments needed to evaluate Atrogin-1 and MuRF-1 expression in *longissimus dorsi* of space flown mice and the onset of back muscle atrophy, we can only speculate about the gap between the expression of these two genes and the translation of the corresponding transcripts. Further investigations are obviously needed to understand the role of Atrogin-1 and MuRF-1 in the regulation of the phenotype in mouse paraspinal muscle.

We recently investigated the morphological changes induced by 30 days microgravity in mouse *soleus* and *extensor digitorum longus* (EDL) (Gambara et al., 2017): reduced CSAs were observed in type I, IIa, IIb, and IIx myofibers in *soleus* of mice flown aboard of the biosatellite BION-M1 whereas no changes were observed in EDL, thus largely confirming the notion that *soleus*, the postural slow type muscle of lower murine limb, was highly responsive to microgravity unloading as compared to EDL, the fast type murine muscle. Moreover, the analysis of myofiber phenotype showed a myofiber type shift from slow to fast mainly in *soleus* muscle of flown mice (Gambara et al., 2017). Since the myofiber type composition of mouse *longissimus dorsi* is prevalently fast, as in the case of EDL, minor atrophying effect of microgravity on this specific back muscle was expected.

Among the genes linked to the transcriptional regulation that we found differentially regulated after microgravity exposure, Mafb (v-maf musculoaponeurotic fibrosarcoma oncogene family protein B) was found to be highly downregulated. Mafb (named also as Kreisler or Krml) is known to be essential for the hindbrain development and defects in the organization of facial motor neurons were found in Mafb mutant mice (McKay et al., 1997). More recently it has been demonstrated that Mafb has a pivotal role in the development of Duane syndrome, a congenital disorder generated by an aberrant cranial innervation (Park et al., 2016). To our knowledge, a role for Mafb in paraspinal muscle physiology has never been described yet, thus further investigation is needed to elucidate the function of Mafb in this particular context.

Focusing mainly on genes that were validated by real time qPCR, almost all these genes are directly or indirectly linked to insulin signaling and sensitivity in skeletal muscle, highlighting the impact of spaceflight on glucose metabolism in the *longissimus dorsi* back muscle. It is well-known that both spaceflight and bed rest induce a set of subclinical diabetogenic effects in humans, such as insulin resistance, decreased glucose tolerance, increased glucose level in plasma and reduced insulin (Tobin et al., 2002). Recently Rudrappa and co-workers reviewed the potential reciprocal influence of insulin resistance and muscle disuse atrophy (Rudrappa et al., 2016), concluding that further studies are needed to better understand the causative role of insulin resistance in determining the atrophic phenotype in skeletal muscle.

Among the genes differentially regulated in *longissimus dorsi* of spaceflown mice, Lipocalin-2 transcripts (Lcn2) were the most upregulated (Fold change = 11.06, BF vs. BG comparison) found in our study. This gene encodes for a small secreted protein that it is known to be involved in the onset of aging and obesity-induced systemic insulin resistance (Law et al., 2010). However, it has been shown that the phosphorylation of insulin receptor and Akt, and insulin-stimulated glucose uptake were not significantly altered in skeletal muscle of Lcn2 knockout mice compared to wild-type mice. These results may be in part explained by the low basal level of Lcn2 expression in murine skeletal muscle (Yan et al., 2007). On the other hand, Guo and co-workers showed opposite results, that is Lcn2 deficiency potentiated diet-induced insulin resistance in mice (Guo et al., 2010). Interestingly, Lcn2 protein level was found to be increased in sera of individuals following bed rest (Rucci et al., 2015), therefore based on our results we may speculate that Lcn2 protein could be at least in part secreted in a soluble form in blood by skeletal muscle tissue similar to the known myokines.

In our study, we also identified another well-known regulator of cell metabolism, Sestrin 1 (Sesn1), that we found to be upregulated in *longissimus dorsi* of spaceflown mice compared to ground controls. Sestrins are a family of stress activated proteins involved in the metabolism of reactive oxygen species (ROS), in the regulation of autophagy, and in insulin signaling. More in detail, Sestrins increase AMPK activation, consequentially inhibiting mTORC1 activity, known to induce insulin resistance through the inhibition insulin receptor substrates and the consequent reduction of phosphoinositide-3-kinase (PI3K)/Akt signaling (Lee et al., 2013). Moreover, it has been shown that only Sestrin 3 (Sesn 3) was upregulated in *vastus lateralis* of type 2 diabetes patients compared to healthy individuals but its role is more likely to be linked to skeletal muscle differentiation than regulating glucose and lipid metabolism. On the other hand, hydrogen peroxide increased only mRNA level of Sesn 1 and 2 in human myotubes, suggesting that these two isoforms are specifically involved in the response to oxidative stress in skeletal muscle (Nascimento et al., 2013). With regard to p85 alpha PI3K regulatory subunit (Pik3r1), we found upregulation of this transcript in *longissimus dorsi* of the spaceflown mice. This result agrees with the upregulation of Pik3r1 found in *gastrocnemius* of mice exposed to microgravity for 11 days (Allen et al., 2009). PI3K is known to be crucial for insulin-induced glucose transport (Hara et al., 1994) and it has been shown that in bed rest, for example, the glucose transporter GLUT4, Akt signaling, and Glycogen synthase activity are equally reduced (Bienso et al., 2012). Interestingly, Terauchi and co-workers showed an increased insulin sensitivity and increase in glucose transport in skeletal muscle of Pik3r1^−/−^ mice due to the compensatory upregulation of the other two isoforms p55 and p50 alpha (Terauchi et al., 1999). To better understand the role of Pik3r1 in the onset or prevention of insulin resistance in disused skeletal muscle, the expression of all Pik3r1 isoforms should be further investigated in microgravity or other experimental models of unloading.

In the current study, we also found that protein kinase C delta (Prkcd) transcript were significantly upregulated in *longissimus dorsi* of spaceflown mice. This result supports the crucial role of Prkcd in skeletal muscle insulin sensitivity. In fact, an increase in the Prkcd levels related to the aging has been previously described, and the muscle-specific Prkcd knockout improved aging-related decline of glucose tolerance and insulin resistance (Li et al., 2015).

Interestingly, two of the validated genes differentially regulated in *longissimus dorsi* of space-flown mice and linked to insulin resistance, Lcn2 and Sesn1, and another gene coding for Mafb were previously identified as overexpressed genes in both *soleus* and EDL of mice flown for 30 days onboard the BION-M1 biosatellite (Gambara et al., 2017), suggesting that the regulation of only few genes following microgravity exposure may not be strictly muscle specific. On the other hand, microarray analysis of space-flown murine *soleus* and EDL compared to ground controls showed that the larger number of differentially regulated genes was highly muscle specific: only 24 differentially regulated genes were in common between the two functionally different muscles (Gambara et al., 2017). In the present study the tongue muscle was used as reference internal control of microgravity exposure as the tongue represents a visceral skeletal muscle with obviously constant activity load. A typical example of tongue loading is performed during daily nutritional activities in microgravity (i.e., water and food uptake) as recently proposed for mice masticatory muscle (masseter, MA). MA insensitivity to microgravity was explained by the protective effect against mass loss due to continuing chewing-induced loading following 13 days spaceflight onboard the STS-135 Space shuttle (Philippou et al., 2015). Further studies are however needed to show if tongue muscle also shares similar atrophy mechanisms than somatic/appendicular skeletal muscle following long-duration spaceflight.

In summary, a number of genes linked to insulin sensitivity and metabolism of skeletal muscle were found significantly dysregulated in the mouse *longissimus dorsi* back muscle following 30 days of microgravity exposure compared to ground control animals. The apparent absence of robust signs of muscle atrophy in paraspinal muscles following 30 days of microgravity opens new questions on the potential roles of these genes in the onset of peripheral insulin resistance following unloading and its consequences on vertebrate skeletal muscle structure and function. Finally, in the present study microgravity-induced transcriptional adaptation of mouse paraspinal muscles has been investigated by means of global gene expression profiling in space-flown mice, providing the basis for a deeper understanding of the complex mechanisms of molecular adaptation to microgravity in vertebrate back muscles as potential cause of lower back pain and spine destabilization processes following whole body unloading conditions in different clinical settings, bed rest, and spaceflight.

## Author contributions

Conceptualization: DB and MS; Data curation: GG, MS, and UU; Formal analysis: GG, UU, and SC; Funding acquisition: DB; Investigation: MS, DB, and GG; Methodology: GS, MG, UU, and SF; Project administration: DB and MS; Resources: DB; Supervision: HG; Validation: UU and SF; Writing—original draft: GG and DB; Writing—review and editing: GG and MS, DB, and PV.

## Funding

This work was supported from grants of the Department of Economics and Technology of the German Government (BMWi) through the German AeroSpace Board, Deutsches Zentrum für Luft- und Raumfahrt (DLR), e.V. Bonn, Germany (grant # 50 WB821, 1121, and 1421 to DB). The funders had no role in study design, data collection and analysis, decision to publish, or preparation of the manuscript.

### Conflict of interest statement

The authors declare that the research was conducted in the absence of any commercial or financial relationships that could be construed as a potential conflict of interest.

## Supplementary material

Figure S1 **CSA heterogeneity and Myofiber minimum Feret diameter. (A)** comparison of the frequency distribution of CSAs in *longissimus dorsi* of the three experimental groups (Figure S1). **(B)** Quantification of the minimum Feret diameter in *longissimus dorsi* of mice from the three experimental groups (BF, BG, and FC, each *n* = 2) shown as scatter plots.

Table S1 **Quantitative PCR primers and conditions**. HK, Reference genes; bp, expected product size; T°, annealing temperature. ^*^ Sandona et al. (2012) Adaptation of Mouse Skeletal Muscle to Long-Term Microgravity in the MDS Mission. PLoS ONE 7(3):e33232.

Table S2 **Microgravity-induced gene expression changes in the tongue muscle of space -flown vs. ground control mice**. List of genes differentially regulated in the tongue muscle (internal reference control) of BF vs. BG mice, meeting *FDR* < 0.05 and fold change < −2 and > +2 criteria. The comparisons between all the three experimental groups are shown.
