# Supplementary figures and images for: Microgravity-Induced Transcriptome Adaptation in Mouse Paraspinal longissimus dorsi Muscle Highlights Insulin Resistance-Linked Genes
Source: Front Physiol. 2017 May 5;8:279. doi: 10.3389/fphys.2017.00279 (PMC5418220; doi:10.3389/fphys.2017.00279)

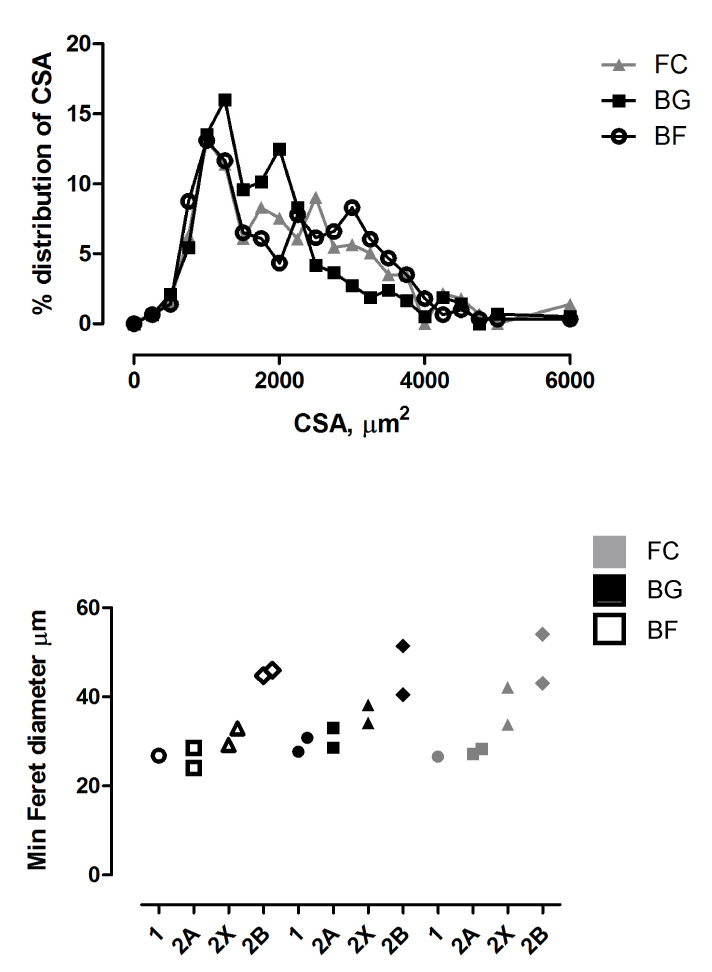

Supplement: Figure S1 — CSA heterogeneity and Myofiber minimum Feret diameter. (A) comparison of the frequency distribution of CSAs in longissimus dorsi of the three experimental groups (Figure S1). (B) Quantification of the minimum Feret diameter in longissimus dorsi of mice from the three experimental groups (BF, BG, and FC, each n = 2) shown as scatter plots. [file Image1.JPEG]
